# Formulation and Testing of Antioxidant and Protective Effect of Hyalurosomes Loading Extract Rich in Rosmarinic Acid Biotechnologically Produced from *Lavandula angustifolia* Miller

**DOI:** 10.3390/molecules27082423

**Published:** 2022-04-08

**Authors:** Matteo Perra, Laura Fancello, Ines Castangia, Mohamad Allaw, Elvira Escribano-Ferrer, José Esteban Peris, Iris Usach, Maria Letizia Manca, Ivanka K. Koycheva, Milen I. Georgiev, Maria Manconi

**Affiliations:** 1 Department of Scienze della Vita e dell’Ambiente, University of Cagliari, 09124 Cagliari, Italy; matteo.perra@unica.it (M.P.); laurafancello2@gmail.com (L.F.); allaw.mohamad.22@gmail.com (M.A.); mlmanca@unica.it (M.L.M.); manconi@unica.it (M.M.); 2 Biopharmaceutics and Pharmacokinetics Unit, Institute for Nanoscience and Nanotechnology, University of Barcelona, 08007 Barcelona, Spain; eescribano@ub.edu; 3 Department of Pharmacy and Pharmaceutical Technology and Parasitology, University of Valencia, 46100 Valencia, Spain; jose.e.peris@uv.es (J.E.P.); iris.usach@uv.es (I.U.); 4 Laboratory of Metabolomics, Department of Biotechnology, Institute of Microbiology, Bulgarian Academy of Sciences, 4002 Plovdiv, Bulgaria; vkoy4eva@abv.bg (I.K.K.); milengeorgiev@gbg.bg (M.I.G.); 5 Department Plant Cell Biotechnology, Center of Plant Systems Biology and Biotechnology, 4002 Plovdiv, Bulgaria

**Keywords:** *Lavandula angustifolia*, lamellar vesicles, hyalurosomes, biotechnologically produced extract, fibroblasts, natural antioxidant

## Abstract

Culture of plant cells or tissues is a scalable, sustainable, and environmentally friendly approach to obtain extracts and secondary metabolites of uniform quality that can be continuously supplied in controlled conditions, independent of geographical and seasonal variations, environmental factors, and negative biological influences. In addition, tissues and cells can be extracted/obtained from the by-products of other industrial cultivations such as that of *Lavandula angustifolia* Miller (*L. angustifolia*), which is largely cultivated for the collection of flowers. Given that, an extract rich in rosmarinic acid was biotechnologically produced starting from cell suspension of *L. angustifolia*, which was then loaded in hyalurosomes, special phospholipid vesicles enriched with sodium hyaluronate, which in turn are capable of both immobilizing and stabilizing the system. These vesicles have demonstrated to be good candidates for skin delivery as their high viscosity favors their residence at the application site, thus promoting their interaction with the skin components. The main physico-chemical and technological characteristics of vesicles (i.e., mean diameter, polydispersity index, zeta potential and entrapment efficiency of extract in vesicles) were measured along with their biological properties in vitro: biocompatibility against fibroblasts and ability to protect the cells from oxidative stress induced by hydrogen peroxide. Overall, preliminary results disclosed the promising properties of obtained formulations to be used for the treatment of skin diseases associated with oxidative stress and inflammation.

## 1. Introduction

*Lavandula angustifolia* Miller (*L. angustifolia*), is a flowering plant of the Lamiaceae family, one of the most famous among the medicinal and aromatic families of plants. It is a native of the Mediterranean area, where it finds its phytosociological optimal environment, expressing a large quantity of secondary metabolites, able to exert biological activities and hence to provide health benefits to humans [1]. Thanks to its phytochemical composition, it is well known worldwide as a powerful aromatic and medicinal herb and is largely used in the toiletry, perfumery, cosmetic and pharmaceutical industries [2,3]. It contains a large amount of volatile (e.g., aromatic compounds) and non-volatile (e.g., phenolic compounds) phytochemicals, which both may have positive effects on human health [3]. Indeed, the main biological or pharmaceutical studies on *L. angustifolia* were performed using the essential oil, which contains many terpenes and volatile compounds, but the plant also contains a high quantity of phenolic compounds such as phenolic acids and flavonoids with well-known antioxidant, anti-inflammatory, antiproliferative and antimicrobial properties [4]. A previous study reported that the phenolics content of the extract of *L. angustifolia* was ~280 µM, corresponding to 0.72 mM Trolox equivalent [5]. Yadikar et al (2018) confirmed that their extract obtained from *L. angustifolia* contained protocatechuic acid, caffeic acid, chlorogenic acid, rosmarinic acid and apigenin, and they also identified seven new phenolic compounds [6]. Adaszyńska-Skwirzyńska and Dzięcioł (2017) found rosmarinic, ferulic and caffeic acid (phenolics), apigenin, luteolin and quercetin (flavonoids) in *L. angustifolia* extracts, confirming that it can be considered a source of antioxidant compounds [7]. Recently, Koycheva et al (2021) obtained a phytocomplex, rich in rosmarinic acid, from *L. angustifolia* by means of biotechnological production, which appeared effective in the treatment of psoriasis [8]. The biotechnological production of phytochemicals from plant-derived cells and organs is a viable, attractive and alternative method for their production as chemical synthesis or plant overcollection, biodiversity depletion and chemical extraction are avoided [9]. Given that, in the last few decades, several studies have focused on the enhancement of the production of plant chemicals by using undifferentiated callus and suspension cultures, which often fail to accumulate the compounds of interest, or better shoot and root cultures, which ensure better production of the same compounds of original plants [10,11]. 

Another strategy aimed at reducing the concentration of phytochemicals for the production of nutraceutical, cosmeceutical and pharmaceutical products, is their formulation in specific nanocarriers, which can load and deliver them through the human organism facilitating the crossing of biological membranes and their accumulation in the desired site [12,13]. Among nanocarriers, phospholipid vesicles represent the most eco-friendly systems due to the natural origin of phospholipids, their structure and composition similar to cell membranes, their biocompatibility and auto-assembling nature, which allow them to be obtained by simple and scalable methods [14]. 

Moreover, the loading of biotechnological phytochemicals on ad hoc formulated phospholipid vesicles is a valuable approach to achieve a double advantage: avoid the depletion of plants and reduce the required concentration of phytochemicals maximizing their efficacy.

Given that, in the present study, the biotechnologically produced extract from *Lavandula angustifolia* Miller, rich in rosmarinic acid, was loaded in hyalurosomes, special phospholipid vesicles, immobilized with hyaluronic acid. The polymer was used at increasing concentrations and the main physico-chemical characteristics and technological properties of the obtained vesicles were evaluated along with their in vitro biocompatibility towards fibroblasts and ability to protect the cells from oxidative damages induced by hydrogen peroxide.

## 2. Results

### 2.1. Extract Bioproduction and Quantification

The extract from *Lavandula angustifolia* Miller cell biomass was obtained as reported below (see Section 4.2). *L. angustifolia* cell suspension was cultivated on supplemented liquid Linsmayer and Skoog (LS) nutrient medium [8]. Following cultivation, the cell suspension was freeze-dried and extracted with a mixture of water and methanol. Then, the extract was filtered, concentrated, freeze-dried and stored until its use. The intensive biosynthesis of the extract began on day 6 of cultivation and the maximum was reached on day 11 [15]. Rosmarinic acid [(R)-a-[[3-(3,4-dihydroxyphenyl)-1-oxo-2Epropenyl]oxy]-3,4-dihydroxy-benzenepropanoic acid] appeared the most abundant compound contained in the extract, being 10.02% of the dried extract powder, whose presence was confirmed by NMR (1D and 2D) and further quantified, by means of HPLC [8]. Hence, the biotechnologically-produced extract from *L. angustifolia* could serve as a source of rosmarinic acid production. Moreover, its high content in this extract suggests that its bioactivity is mainly due to the presence of specific phenolic compounds [16].

### 2.2. Vesicle Preparation and Characterization

A preformulation study was carried out using an increasing concentration of extract (5, 10, 20 mg/mL), different kinds and amounts of phospholipids and biopolymers. Finally, P90G (195 mg/mL) was selected as the more suitable phospholipid, which has been combined with sodium hyaluronate at different concentrations (0.05, 0.1 and 0.2 mg/mL). The composition of the vesicles is reported in Table 1.

The average diameter, polydispersity index and surface charge of vesicles have been measured (Table 2). 

The 0.05% hyalurosomes containing both the lowest concentration of the extract (5 mg/mL) and hyaluronan (0.5 mg/mL) were the smallest (~123 nm) with the lowest polydispersity index (~0.26), while 0.2% hyalurosomes prepared with the highest concentration of the extract (20 mg/mL) and hyaluronan (2 mg/mL) were the largest (~161 nm) and highly polydispersed as the polydispersity index was ~0.32. When 10 and 20 mg/mL of the extract were loaded in 0.05% hyalurosomes, the size slightly increased up to ~134 nm in comparison with 0.05% hyalurosomes containing the lowest amount of the extract, while the polydispersity index remained unchanged, disclosing a good homogeneity of vesicular dispersion using the lowest concentration of hyaluronan. The size and polydispersity index of 0.1% hyalurosomes was higher than those of 0.05% hyalurosomes except when 20 mg/mL of the extract was loaded. The size and polydispersity index of 0.2% hyalurosomes loading 5 mg/mL of the extract were similar to that of 0.05% hyalurosomes loading 10 or 20 mg/mL of the extract. However, when 10 and 20 mg/mL of the extract were loaded the size of 0.2% hyalurosomes significantly increased (~158 nm). The zeta potential was less negative when the hyaluronan concentration increased, confirming its positioning also on the vesicle surface. The loading of the extract affected the zeta potential values to a lesser extent than the polymer. The amount of extract effectively incorporated in the hyalurosomes was ~100% (*p* > 0.05 among the values of this group), irrespective of the used concentrations of both the extract and hyaluronan. 

The antioxidant efficacy of *L. angustifolia* extract was previously demonstrated, so that the free radical scavenging activity of formulations was assessed by means of the DPPH colorimetric assay and expressed as a function of Trolox equivalents [17]. The Trolox equivalents of prepared formulations were not directly affected by the concentration of the extract, more specifically, the hyalurosomes loading 5 mg/mL of the extract had the lowest values, ~1.71 mg/mL of sample for 0.05% and 0.2% hyalurosomes, or ~1.54 mg/mL of sample for 0.1% hyalurosomes. Hyalurosomes loading 10 mg/mL reached the highest values, ~1.81 mg/mL of sample for 0.05% and 0.2% hyalurosomes or ~1.93 mg/mL of sample for 0.1% hyalurosomes. The Trolox equivalents of hyalurosomes loading 20 mg/mL decreased, being ~1.77 mg/mL of sample, denoting a quenching of antioxidant activity probably related to the saturation of solution.

Compared to the DPPH colorimetric test the FRAP assay showed a direct correlation between the concentration of the extract and its activity (Table 3). Indeed, the hyalurosomes loading 5 mg/mL of the extract showed the lowest values irrespective of the percentage of sodium hyaluronate; while hyalurosomes loading 20 mg/mL of the extract showed the highest FRAP values. These differences between the two performed assays may be due to the different structure–activity relationships between polyphenols and assay results [18].

Overall, 0.1% hyalurosomes loading 10 mg/mL of the extract had the highest value of Trolox equivalents. 

**Figure 1:**
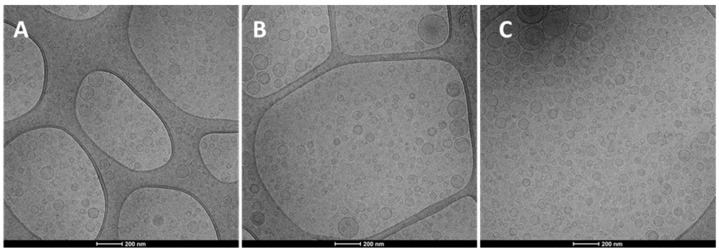
Representative Cryo-TEM images of 0.05% hyalurosomes loading 5 mg/mL of extract (**A**); 0.1% hyalurosomes loading 10 mg/mL of extract (**B**); and 0.1% hyalurosomes loading 20 mg/mL of extract (**C**).

The Cryo-TEM observation revealed that the structure and morphology of prepared hyalurosomes were very similar irrespective of the used concentration of both the extract and hyaluronan (Figure 1). Vesicles were spherical and mostly uni- and oligo-lamellar. Considering all the measured parameters, the polydispersity index seems to be the most critical, because the value of the polydispersity index ≥0.3, indicated a polydispersed sample in which sometimes small amounts of very large vesicles, unstable and capable of quickly precipitating, are present. The highest value of the polydispersity index (≥0.3) was found for dispersions of hyalurosomes prepared with higher concentrations of the extract (10, 20 mg/mL) and hyaluronan (0.1, 0.2 mg/mL). Given that, and according to the highest Trolox equivalents, the intermediate concentration of both payload and polymer (i.e., 0.1% hyalurosomes loading 10 mg/mL of extract) was selected for further studies.

### 2.3. Biocompatibility of Formulations 

**Figure 2:**
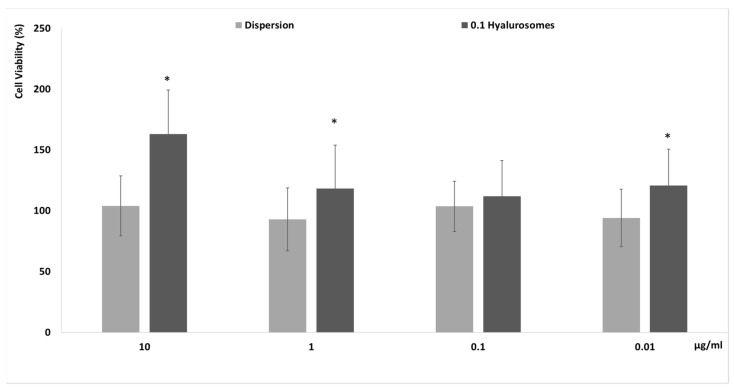
Cell viability of fibroblasts treated for 48 h with extract in dispersion or loaded in hyalurosomes diluted to reach 10, 1, 0.1, 0.01 μg/mL of extract. Data are reported as mean values (*n* = 9) ± standard deviations (error bars) of cell viability expressed as the percentage of untreated cells (100% of viability). Symbol (*) indicates that the viability of cells treated with 0.1 hyalurosomes is statistically different from that of cells treated with the extract in dispersion (*p* < 0.05).

The in vitro biocompatibility of novel formulations has to be proven before the evaluation of efficacy to be sure of their effective safety. Firstly, specific tests that are reliable and predictive of the behavior that can occur in vivo must be completed in vitro by using cells. In this study, the evaluation of the biocompatibility of the selected formulation was performed in vitro using fibroblasts (3T3), which can be considered the most representative cells of the dermis, as these formulations have been specifically tailored to be capable of reaching the deeper skin layers after their application on the skin surface [19]. The metabolic activity of cells was measured by MTT test, which is considered an indicator of their viability. Four different concentrations 0.01, 0.1, 1 and 10 µg/mL, have been selected as a suitable range for preliminary in vitro testing. Indeed, considering the possible dilution that can occur in vivo, 10 μg/mL of the extract seemed to be reliably the maximum amount effectively capable of reaching the deeper strata of the skin. The extract in dispersion at the same concentrations has been used as reference aiming at understanding the effect of both the extract and carriers on cell viability (Figure 2). The cell activity was ~100%, upon incubation with the extract in dispersion, irrespective of the used dilutions, confirming the high biocompatibility of the components of the extract. Their loading in hyalurosomes increased the metabolic activity and viability of cells, which reached ~150%. This positive effect of extract-loaded hyalurosomes is probably due to the beneficial properties of sodium hyaluronate, which may be able to stimulate the proliferation of different cells, as previously reported [19,20].

### 2.4. Protective Effect of the Formulations against Damages Induced by Hydrogen Peroxide 

**Figure 3:**
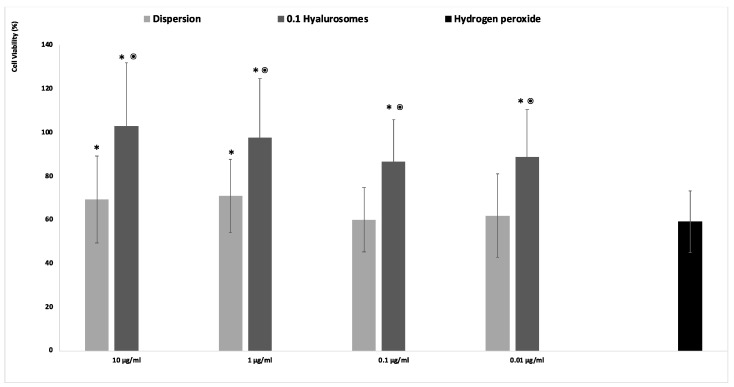
Viability of fibroblasts stressed with hydrogen peroxide and protected with *Lavandula angustifolia* extract in dispersion or loaded in hyalurosomes properly diluted to reach 10, 1, 0.1, 0.01 μg/mL of extract. Data are reported as mean values (*n* = 9) ± standard deviations (error bars) of cell viability expressed as the percentage of untreated cells (100% viability). Symbol (*) indicates that the viability of the cells treated with the extract in dispersion and loaded in 0.1 hyalurosomes are statistically different from that of cells treated with hydrogen peroxide (*p* < 0.05); symbol (^⦿^) indicates that the viability of the cells treated with 0.1 hyalurosomes is statistically different from that of cells treated with the extract in dispersion (*p* < 0.05).

The acute exposition of cells to hydrogen peroxide caused cell apoptosis and death, due to the generation of dangerous reactive oxygen species [21]. The in vitro treatment of cells with this damaging agent is usually used to evaluate the ability of drugs to prevent or inhibit the onset of oxidative damage, which in turn accelerates premature senescence and dangerous modification of cells. Given that, in this study, the fibroblasts were stressed with hydrogen peroxide and simultaneously protected with the extract in dispersion or loaded in hyalurosomes (Figure 3). The activity of cells stressed with hydrogen peroxide decreased up to ~60%, that of cells stressed and protected with the extract in dispersion was the same (~60%) when lower concentrations of the extract (0.1 and 0.01 mg/mL) were used, and become slightly higher ~70% using 1 and 10 µg/mL of the extract. This indicates that at higher dilutions the extract was ineffective in counteracting the reactive species generated by hydrogen peroxide, being unable to avoid the death of cells. When the extract was loaded in hyalurosomes, its ability to inhibit the formation and spread of free radicals was enhanced as the cell activity was ~120%, irrespective of the used dilution. The higher efficacy of the extract loaded in hyalurosomes, also at lower dilutions, can be due to its higher internalization inside the cells thanks to the ability of phospholipid vesicles to fuse and cross the cell membrane [22].

## 3. Discussion

The skin is the largest organ, which acts as an effective barrier capable of protecting, isolating, enclosing and supporting the human body. Being the first interface with the external environment, it is constantly subjected to chemical, physical and mechanical insults, which can affect its metabolism and functions [23]. These factors or their metabolites can, directly or indirectly, lead to the overproduction of several reactive oxygen species (ROS) [23]. A low quantity of ROS is naturally produced during the normal aerobic metabolism of cells and physiologically neutralized by the endogenous antioxidant mechanism [24]. However, an uncontrolled and prolonged overproduction of ROS at skin level, is firstly involved in accelerated ageing, followed by the pathogenesis or progression of several human skin disorders including psoriasis, dermatitis and cutaneous neoplasia [25,26]. The major consequence associated with skin aging is a modification of its mechanical properties, predominantly related to an alteration of both production and structural organization of collagen and elastin and a decreasing of the density of the extracellular matrix, which are associated with a higher stiffness of dermal fibroblasts [27]. Furthermore, long-term exposure to ROS causes DNA damage, which may induce cell modification and neoplasia formation [28]. Given that, the use of antioxidant molecules capable of preventing or at least slowing the oxidative processes is considered the most promising strategy for human health. Among the antioxidant molecules, the natural ones and especially those extracted from plants seem to be the most effective. Indeed, plants are rich in polyphenolic bioactives, which can exert their activity by reducing or inhibiting free radicals’ production, thanks to their ability to transfer hydrogen atoms from hydroxyl group [29].

As is well known, the extract obtained from *Lavandula angustifolia* is rich in antioxidant bioactives, especially rosmarinic acid, which is the main element responsible for its beneficial effects. The mechanism responsible for the antioxidant activity of rosmarinic acid has been already investigated and confirmed by Joardar et al [16]. Indeed, the treatment of cells with rosmarinic acid significantly counteracted the CdCl_2_-induced ROS production and reduced NO and NADPH oxidase levels, H_2_O_2_ content, lipid peroxidation and protein carbonylation [29]. In addition, rosmarinic acid significantly downregulated nitric oxide and PGE_2_ production induced by IL-1b in chondrocytes [30].

Despite the promising properties of the bioactives contained in *Lavandula angustifolia* extract, their activity is often hampered because of their instability, so that their incorporation/protection into nanocarriers may represent the most promising approach. In light of this, in the present study, special vesicles, so-called hyalurosomes, have been formulated for the effective incorporation of the extract rich in rosmarinic acid and biotechnologically produced from *L. angustifolia* and especially aimed at protecting fibroblasts from oxidative stress. Hyalurosomes were selected because of their peculiar properties. Indeed, the acidic chains of hyaluronan at pH > 3 are highly ionized forming strong intermolecular interactions, with the consequent development of a structured network having particular viscoelastic properties and capable of immobilizing the vesicles. These systems are also capable of avoiding the leakage of the formulation from the skin thus facilitating both the interaction with skin components and the passage of the bioactives into and through the skin [31]. In addition, hyaluronan itself has healing properties as its abilities to promote skin repair and wound healing are well known [32]. In a preformulation study, the concentration of the extract and hyaluronan, capable of leading the formation of small, stable and efficient vesicles, were selected. It was found that 0.1% hyalurosomes had a mean diameter of ~140 nm and were monodispersed, being the polydispersity index ~0.28, thus were ideal for skin delivery, indeed, Castangia et al. (2015) used similar hyalurosomes (~100 nm) for the delivery of another extract, while Abruzzo et al. (2020) obtained optimal results in the skin delivery of glycyrrhetinic acid using larger hyalurosomes, ~355 nm [20,33]. In addition, the selected formulation had the highest Trolox equivalents, confirming it as having the best ability to neutralize ROS [34,35]. On the contrary, no studies have been performed on the incorporation of *L. angustifolia* extract in hyalurosomes or other phospholipid vesicles neither has its efficacy been tested for the treatment of skin diseases connected with oxidative stress and aging. In the light of this, for the first time in this study, the ability of *L. angustifolia* extract-loaded hyalurosomes to protect skin fibroblasts from oxidative stress was confirmed by in vitro studies. The protective effect was ensured by the loading of the extract in hyalurosomes, which can interact, to a better extent than the extract in dispersion, with the cell membrane facilitating the phytochemical internalization in the cytoplasm, where they can terminate free radical chain reactions, capture free radicals or convert radicals into less active forms, preventing cell damages [36]. Indeed, the extract loaded in hyalurosomes avoided cell death caused by hydrogen peroxide, thus ensuring the maintenance of healthy condition at all the tested dilutions (from 10 to 0.01 μg/mL of the extract), while the free extract (unloaded) had a low protective activity which was slightly evident only when using the higher concentrations. This indicates that by using the hyalurosomes it is possible to reduce the amount of extract required to obtain the desired biological effect/activity. This is an important result, because among the main limitations of using commercial extracts to prepare herbal formulations, the high cost and the effective content of phytochemicals are the most difficult to overcome. The loading of the biotechnological extract of *L. angustifolia* in hyalurosomes can eventually help to solve these limitations because it allows a reduction in the amount of extract needed, which, when biotechnologically produced, has a constant composition irrespective of environmental or seasonal conditions and avoids the depletion of natural resources. 

## 4. Materials and Methods

### 4.1. Materials

Phospholipon 90G (P90G) was purchased from Lipoid AG (Cologne, Germany) with the support of its Italian agent AVG srl (Milan, Italy). Sodium hyaluronate was purchased from Pentapharm DSM Nutritional Products AG (Aesch, Switzerland). All the chemical products and solvents of analytical grade were purchased from Sigma-Aldrich (Milan, Italy). Cell medium, foetal bovine serum, penicillin, streptomycin and all the other reagents and plastic for cell culture were purchased from Life Technologies Europe (Monza, Italy).

### 4.2. Bioproduction of Extract

The *Lavandula angustifolia* Miller cell suspension culture was cultivated, as previously reported [15], on liquid Linsmayer and Skoog (LS) nutrient medium, supplemented with 30 g/L sucrose and 0.2 mg/L of 2,4-dichlorophenoxyacetic acid, at 26 °C, in the dark, on a rotary shaker at 100 rpm. Further, *L. angustifolia* cell biomass was harvested, dried and extracted with 50% aqueous methanol (*v*/*v*) under sonication, at room temperature, for 20 min [8]. The obtained extract was filtered, vacuum concentrated at 40 °C, freeze-dried and stored at −20 °C until its use.

### 4.3. Ability of Extract to Scavenge Free Radicals 

The antioxidant potential of the extract was assessed by measuring its ability to scavenge the DPPH (1,1-diphenyl-1-picrylhydrazil) radicals. Its ethanolic solution (1 mg/mL) was firstly diluted 1:50 to reduce the color of the solution and then 20 µL of the diluted solution was mixed with 1980 μL of DPPH methanolic solution (40 µg/mL), and incubated for 30 min, at room temperature, in the dark. Then, the absorbance was measured at 517 nm against blank. The antioxidant activity was calculated according to the following formula:

antioxidant activity (%) = [(ABSDPPH − ABSsample)/ABSDPPH] × 100.(1)

A calibration curve using Trolox (6-hydroxy-2,5,7,8-tetramethylchroman-2-carboxylic acid) at different concentrations (0–0.010 mg/mL) was built and used as a reference and expressed as mg of Trolox equivalent/g of dry powder extract. All the experiments were performed in triplicate.

### 4.4. Ferric Reducing Antioxidant Assay

The ferric reducing antioxidant assay (FRAP) is based on the reduction of ferric 2,4,6-tris(2-pyridyl)-1,3,5-triazine [Fe(III)-TPTZ] to the blue colored ferrous complex by antioxidants in acidic medium. The reduction is monitored at 593 nm by means of spectrophotometric measurements. A total of 10 µL of the tested sample was added to 300 µL of freshly prepared reagent (0.3123 g TPTZ, 0.5406 g FeCl_3_·6H_2_O in 100 mL acetate buffer pH 3.6). Quantitative analysis was performed using the external standard method (ferrous sulphate, 0–2000 µM), correlating the absorbance (λ = 593 nm) with the concentration. The results were expressed as µmol of Fe^2+^ per ml of sample.

### 4.5. Vesicle Preparation 

Table 1 shows vesicle composition. Briefly, P90G (195 mg/mL) and the extract at increasing concentrations (5, 10, 20 mg/mL) were weighed in a glass vial and hydrated with an aqueous dispersion of sodium hyaluronate (0.5, 1 or 2 mg/mL). Dispersions were left hydrating for 2 h, to promote the swelling of the phospholipid, and then sonicated (3 s on and 2 s off, 20 cycles; 14 microns of probe amplitude) with a high intensity ultrasonic disintegrator (Soniprep 150, MSE Crowley, London, UK) to obtain small and homogenous vesicles. Empty formulations were also prepared and used as references.

### 4.6. Vesicle Characterization 

Cryogenic electron transmission microscopy (cryo-TEM) analyses were performed by using a Tecnai F20 TEM (FEI Company). Briefly, a thin aqueous film was formed on a glow-discharged holey carbon grid and vitrified by plunging into ethane, using a Vitrobot (FEI Company, Eindhoven, The Netherlands), which was then observed in a low dose mode, at 200 kV and at a temperature ca. ~−172 °C.

The average diameter and polydispersity index of the vesicles were determined by photon correlation spectroscopy by using a Zetasizer ultra (Malvern Instruments, Worcestershire, UK). The Zetasizer ultra was also used to measure the surface charge of vesicles (zeta potential) measuring their electrophoretic mobility in dispersion with the mixed-mode measurement-phase analysis (M3-PALS). Each sample was diluted (1:100) to be optically clear and to avoid the attenuation of the laser beam by the particles along with the reduction of scattered light that can be detected.

The entrapment efficiency was calculated as the percentage of antioxidant activity of vesicle dispersions measured before and after their purification from the unentrapped extract by dialysis. The vesicle dispersions (1 mL) were loaded into dialysis tubes (Spectra/Por^®^ membranes, 12–14 kDa MW cut-off, 3 nm pore size; Spectrum Laboratories Inc., Breda, The Netherlands) and maintained at room temperature in one liter of water for 2 h, refreshing with water after 1 h. The antioxidant activity of formulations before and after the dialysis was measured by means of the DPPH colorimetric test. 

### 4.7. In Vitro Cytotoxicity of Formulations 

Primary mouse embryonic fibroblasts (3T3; ATCC collection, Manassas, VA, USA) were grown as monolayers in 150 cm^2^ flasks, incubated with 100% humidity and 5% CO_2_ at 37 °C. Phenol red-free Dulbecco’s Modified Eagle Medium (DMEM) with high glucose, supplemented with 10% fetal bovine serum, penicillin and streptomycin, was used as culture medium. Cells were seeded into 96-well plates (5 × 10^4^ cells/well). At 24 h, cells were treated for 48 h with *L. angustifolia* extract in dispersion (reference) or loaded in hyalurosomes. Samples were diluted with the cell medium to reach different extract concentrations (10, 1, 0.1, 0.01 µg/mL). At the end of the experiments, the cells were washed with warmed phosphate buffer solution and their activity was measured using the MTT [3(4,5-dimethylthiazolyl-2)-2,5-diphenyltetrazolium bromide] colorimetric assay. MTT solution (100 µL 0.5 mg/mL in PBS, final concentration) was added to each well, and cells were incubated for 3 h. After that, the formed formazan crystals were dissolved in 100 µL of dimethyl sulfoxide and their concentration was spectrophotometrically quantified at 570 nm by using a microplate reader (Synergy 4, Reader BioTek Instruments, AHSI S.P.A, Bernareggio, Italy). Results are shown as percent of cell viability in comparison with non-treated cells (100% viability).

### 4.8. In Vitro Protective Effect of Formulations against Oxidative Damage in Fibroblasts

The ability of the extract to protect fibroblasts from damages induced by hydrogen peroxide was evaluated. Cells were seeded in 96-well plates (5 × 10^4^ cells/well) and incubated at 37 °C in 5% CO_2_ for 24 h. Cells were stressed with hydrogen peroxide (30% diluted 1:40,000 *v*/*v* with medium) and treated for 4 h with the extract in dispersion or loaded in vesicles suitably diluted with cell medium to reach 10, 1, 0.1, 0.01 µg/mL of the extract. Cells stressed with hydrogen peroxide and untreated were used as negative control, healthy cells unstressed and untreated were used as positive control. At the end of each experiment, the cells were washed with phosphate buffer solution and the MTT assay was used to assess the viability, as reported above. 

### 4.9. Statistical Analysis of Data

Results are expressed as the mean ± standard deviation. Analysis of variance (ANOVA) was used for multiple comparisons of means, and the Tukey’s test and Student’s *t*-test were performed to substantiate differences between groups using XL Statistics for Windows. The differences were considered statistically significant for *p* < 0.05.

## 5. Conclusions

Formulation study confirmed that the biotechnologically produced extract of *L. angustifolia* can be effectively incorporated in hyalurosomes, specifically tailored for skin administration, thus reducing the concentration required to obtain the biological effect. In addition, the use of a biotechnologically produced extract, significantly avoids plant depletion or cultivation, improving its sustainability and carbon footprint. The selected formulation could be considered as a potential system to protect fibroblasts against the damaging environmental factors, as the results indicate that it effectively avoided cell death and restored health conditions.

## Figures and Tables

**Table 1:** Composition of *L. angustifolia* extract loaded hyalurosomes.

	Extract, mg/mL	P90G, mg/mL	Hyaluronan, mg/mL	Water, mL
0.05% hyalurosomes	5	195	0.5	2
0.05% hyalurosomes	10	195	0.5	2
0.05% hyalurosomes	20	195	0.5	2
0.1% hyalurosomes	5	195	1	2
0.1% hyalurosomes	10	195	1	2
0.1% hyalurosomes	20	195	1	2
0.2% hyalurosomes	5	195	2	2
0.2% hyalurosomes	10	195	2	2
0.2% hyalurosomes	20	195	2	2

**Table 2:** Mean diameter (MD); polydispersity index (PI); zeta potential (ZP); entrapment efficiency (EE) and Trolox equivalents (TE). Mean values ± standard deviations are reported (*n* = 6). Same symbol (*, °, ^§^, ^#^) indicates the same value (*p* > 0.05).

	Extract, mg/mL	MD, nm	PI	ZP, mV	EE, %
0.05% hyalurosomes	5	° 123 ± 5	0.26 ± 0.01	−21 ± 1	99 ± 1
0.05% hyalurosomes	10	^#^ 133 ± 4	0.26 ± 0.01	−24 ± 1	100 ± 1
0.05% hyalurosomes	20	^#^ 135 ± 3	0.27 ± 0.02	−27 ± 1	100 ± 1
0.1% hyalurosomes	5	* 142 ± 4	0.30 ± 0.03	−21 ± 1	106 ± 3
0.1% hyalurosomes	10	* 141 ± 5	0.28 ± 0.01	−24 ± 1	101 ± 1
0.1% hyalurosomes	20	*^#^ 137 ± 20	0.30 ± 0.04	−26 ± 1	101 ± 1
0.2% hyalurosomes	5	^#^ 130 ± 2	0.26 ± 0.01	−23 ± 1	100 ± 1
0.2% hyalurosomes	10	^§^ 157 ± 4	0.28 ± 0.01	−25 ± 1	100 ± 1
0.2% hyalurosomes	20	^§^ 161 ± 12	0.32 ± 0.04	−26 ± 1	100 ± 1

**Table 3:** Antioxidant activity of *L. angustifolia* extract in dispersion or loaded in hyalurosomes, expressed as Trolox equivalents (TE) and µmol Fe^2+^ (FRAP). Mean values ± standard deviations are reported (*n* = 6).

	Extract mg/mL	TE mg/mL of Sample	FRAP µmol Fe^2+^/mL of Sample
5-Dispersion	5	1.72 ± 0.08	228.69 ± 22.40
10-Dispersion	10	1.85 ± 0.06	368.02 ± 49.29
20-Dispersion	20	1.76 ± 0.03	554.53 ± 35.80
0.05% hyalurosomes	5	1.71 ± 0.04	200.97 ± 16.61
0.05% hyalurosomes	10	1.81 ± 0.01	325.83 ± 3.95
0.05% hyalurosomes	20	1.76 ± 0.02	531.63 ± 7.89
0.1% hyalurosomes	5	1.54 ± 0.07	223.18 ± 13
0.1% hyalurosomes	10	1.93 ± 0.01	362.68 ± 5.43
0.1% hyalurosomes	20	1.80 ± 0.02	533.18 ± 1.30
0.2% hyalurosomes	5	1.72 ± 0.02	249.19 ± 16.13
0.2% hyalurosomes	10	1.80 ± 0.01	426.58 ± 7.89
0.2% hyalurosomes	20	1.74 ± 0.01	600.18 ± 4.14

## Data Availability

Not applicable.

## Sample Availability

Samples of the compounds are not available from the authors.
